# Harm-Benefit Analysis May Not Be the Best Approach to Ensure Minimal Harms and Maximal Benefits of Animal Research—Alternatives Should Be Explored

**DOI:** 10.3390/ani10020291

**Published:** 2020-02-12

**Authors:** Yoram Gutfreund

**Affiliations:** Department of Neurobiology, Rappaport Faculty of Medicine and Research Institute, Technion—Israel Institute of Technology, Haifa 3100000, Israel; yoramg@technion.ac.il

**Keywords:** the 3Rs, cost-benefit, animal research, animal experiments, IACUC

## Abstract

**Simple Summary:**

In this commentary, I discuss the caveats of incorporating a harm-benefit analysis (HBA) as part of the ethical evaluation of animal research. I argue that employing HBA can lead to decisions that are deleterious for research as well as animal welfare. I therefore call on policy-makers to reconsider the placement of HBA jargon in the guidelines and support an alternative guideline that is both practical and ethical. I based this argument on theoretical considerations but also on my years of experience as head of an Institutional Animal Care and Use Committee (IACUC) and as a representative in the Israeli Council of Animal Experimentation.

**Abstract:**

Using animals in scientific research is commonly justified on the utilitarian basis that the benefits of scientific progress to human health and society exceed by far the harm inflicted on animals. In an attempt to ensure that this is indeed the case for every research project, legislation and guidelines increasingly demand the application of harm-benefit analysis (HBA) as part of the approval process of animal research protocols. The ethical principle of HBA asserts that the costs of an action should be weighed against the expected benefits. Any action that may inflict harm can only be approved if it is associated with a greater benefit. This principle is intuitively appealing but how to use it as a practical rule for ethical decisions is a difficult question. The main difficulty is that the future benefits of most scientific research are unmeasurable, unpredictable and are not manifested at the level of the individual project. Applying HBA in such cases may impede scientific progress by inducing a bias against basic research. Moreover, it can lead to the toleration of unnecessary harm to animals in research. Given these caveats of HBA, I call policy-makers to reconsider the place of HBA in animal research. Instead, I support an alternative guideline which is based on replacing the HBA principle (that the expected benefits of the research must exceed the harms caused to the animals) with two independent but mutually necessary principles: (1) any research using an animal must carry a benefit for society and (2) the harm inflicted to an animal in an experiment must be minimal and scientifically justified. I argue that rigorous harm-analysis, which is not weighted against obscure benefits, can increase the over-all benefits of research while reducing the harms to animals.

## 1. Introduction

A common criticism directed at animal experiments is that, although scientists say that animal research is critically important for saving and improving human lives, most of the research is basic research not aimed at saving lives. Exploiting animals purely for the gratification of the scientist’s personal curiosity is not justified [1]. This criticism is efficient and powerful, influencing public opinion, policy-makers and legislators, leading to the popular demand to ban animal experiments in research that is curiosity-driven. 

In part, as a response to this political pressure, the idea of applying harm-benefit analysis (HBA) as part of the approval process of animal research is becoming increasingly more accepted by both scientists and policy-makers [2,3]. The ethical principle of HBA asserts that the costs of an action should be weighed against the expected benefits. Any action that may inflict harm can only be justified ethically if it is associated with a greater benefit. This is a utilitarian principle that is intuitively appealing. But how can it be used as a practical guideline for ethical decisions? In some simple cases, the harms and benefits can be positioned unequivocally on the same scale. For example, in human pharmaceutical research, the risk of administering a new drug to a patient can be contrasted against the benefits of possible recovery from the disease. But animal research rarely falls into such clear rubrics of harms and benefits. In most cases, the benefits are manifested indirectly by incorporating knowledge from multiple researches. The harms, on the other hand, are directly inflicted to the animals at the single research level. How to decide when harms outweigh the benefits or vice versa is therefore not at all trivial. This raises the controversy: should HBA be applied as a guideline for animal research committees (IACUC—Institutional Animal Care and Use Committee, will be used here as a general term for any ethical committee approving animal use in research) and if yes, how? [2,4,5]. The National Research Council (NRC) guide for the care and use of laboratory animals does not imply HBA as a mandatory guideline for the assessment of animal research [6]. On the other hand, the United Kingdom (UK) Animals Act and the European directive state that the evaluation of each research study should include a harm-benefit analysis [7,8].

“In carrying out the evaluation of a programme of work the Secretary of State must—, (d) carry out a harm-benefit analysis of the programme of work to assess whether the harm that would be caused to protected animals in terms of suffering, pain and distress is justified by the expected outcome, taking into account ethical considerations and the expected benefit to human beings, animals or the environment;”

*UKSection 5(3) of the Animals (Scientific Procedures) Act 1986.*

“The likely harm to the animal should be balanced against the expected benefits of the project… The project evaluation shall consist in particular of the following: d) a harm-benefit analysis of the project to assess whether the harm to the animals in terms of suffering, pain and distress is justified by the expected outcome taking into account ethical considerations and may ultimately benefit human beings, animals or the environment.”

*Directive 2010/63/EU of the European Parliament and the Council of 22 September 2010 on the protection of animals used for scientific purposes.*

However, the guidelines do not specify a clear explanation as to how this should be done. This leaves room for interpretations, resulting in different committees finding diverse ways and solutions on how to deal with the difficulty of incorporating the principles of HBA in their decisions [9,10,11,12].

Over the years, at least three formal working groups have been established to formulate explicit guidelines [13,14,15]. The working groups addressed the issues at length in their reports and discussed several models and algorithms for HBA. But in the end, they did not provide general and clear rules on how to apply HBA. It is becoming apparent that employing HBA in the approval process of animal research is more difficult than anticipated. 

The main problem is that most animal research, unlike human research, is basic research, where the aim is to acquire scientific knowledge [16]. There is no doubt that scientific knowledge affords unequivocal benefits but these benefits are unmeasurable and unpredictable. It is not at all clear how is it possible to assess if the expected outcomes of the research justify the harm (as required by the European act) when the expected outcome is knowledge and not any measurable benefit. Thus, on the one hand, legislations call IACUCs to carry out HBA but on the other hand, objective HBA is unfeasible in most examples of animal research. Below I argue that in such situations, adapting HBA principles can lead to unwanted biases against basic research and to the toleration of unnecessary harm to animals in research. 

## 2. HBA May Compromise Scientific Progress

The immense body of knowledge on the human body, which is the basis of modern medicine, has been acquired by numerous scientists performing vast numbers of experiments, most of them using animals. Evaluating each of these experiments independently, it is hard to understand the importance but combined, they form a lifesaving body of knowledge [17]. Such gradual advances in knowledge are achieved if scientists are given academic freedom to let their scientific curiosity guide the questions they ask. It has been long and widely accepted that for the sake of science and society, scientists should be granted academic freedom in their quest for knowledge [18]. 

At this point of the discussion, it is important to emphasize that the value of academic freedom does not conflict with the value of ethical conduct of research. Academic freedom is about *what* to study and ethics is about *how* to study. In other words, academic freedom is the freedom to choose the scientific questions but it does not grant the freedom to act unethically [19]. 

Granting IACUCs the mandate to apply HBA implies that there are research programs that are more important than others and it is the committees’ role to police the research aims according to potential importance (expected benefit). But this is paradoxical because, as explained above, for science to be beneficial, research aims cannot be policed. We should oppose external control over research questions in all fields of science. It is obviously destructive that a government official will evaluate research in a history department to decide which research proposals are patriotic enough to receive governmental funding and which are not. Similarly, society should be extremely careful in granting a committee the authority to decide which life science research is beneficial enough to receive governmental permits and which is not.

**Figure 1:**
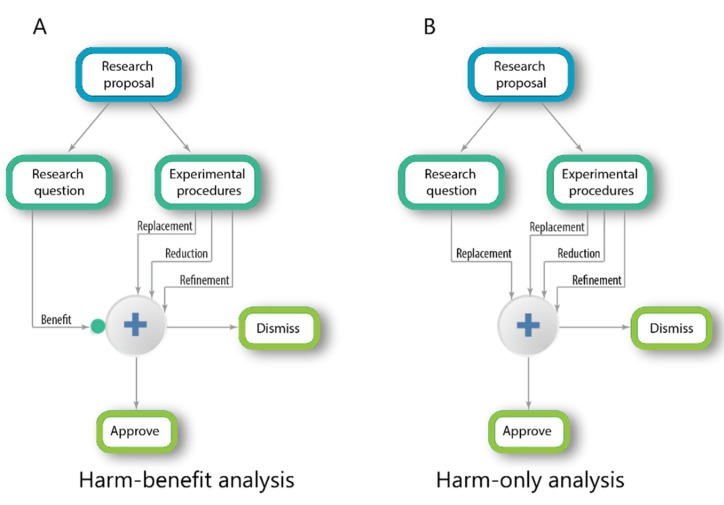
Harm-benefit analysis vs. harm-only analysis.

The diagram in Figure 1A illustrates a classical HBA procedure. The research proposal is first divided into the research question, which includes the research aims and into the research program, which includes the experimental procedures, the animal species, the number of samples and so forth. The research program is evaluated according to the well-established ethical principles of Replacement, Reduction and Refinement (the three Rs [20]). In parallel, the potential or expected benefits of the research aims are evaluated and this, in turn, modulates the ethical criteria [14]. If the score is low, the threshold is raised, making it more difficult to approve. 

The problem with such a procedure is that somebody must evaluate the expected benefit of the research question and categorize research as being important or not. Who will do this and how? Any research that is commonly categorized as being potentially beneficial to human health cannot be arrived at without carrying out multiple other research studies that are usually not categorized as being important. It is extremely difficult to predict or follow the outcome of an individual research project. Therefore, sorting research according to importance is inherently subjective. Consequently, if a committee insists (or is forced) to evaluate the expected benefits, it is not the true benefit of the research that governs the committee’s decisions but the publicly perceived benefit, which is strongly influenced by public relations and popularity. Generally, research that is directly linked to well-known diseases is likely to be preferred over research whose link with human life is not immediately obvious [5]. Yet the *true* benefit of the latter may easily exceed the benefit of the former. 

The argument above that HBA in IACUC discussions may result in a bias against basic research is not a mere theoretical concern. It is a reality, particularly in institutes where HBA regulations are being strongly implemented [21]. Thus, attempts to admit HBA into IACUC considerations paradoxically carry the risk of reducing the overall benefits of research. If this is the price to pay for ensuring reduced harm being caused to animals in research, the risk might be acceptable. But, in my experience, tying the harm with the expected benefits not only does not reduce overall harm but may increase it.

## 3. HBA May Compromise Animal Welfare

The meaning of employing HBA is that animal welfare considerations are not applied equally to all-researches that are favored as beneficial are given permits to do more harm to animals compared to research being carried out in other labs. Thus, the justification of excessive harm is a risk of HBA. However, in animal research, this risk is meant to be balanced by the principle of Refinement (one of the 3Rs). The principle of refinement, that harm to animals should be the minimal necessary to achieve the scientific goals, is supposed to be applied equally to all programs, independent of their evaluated benefits [14]. Thus, in theory harm is kept at the minimal possible level to start with. From here, HBA can only reduce total harm. However, this assumes an ideal situation where every research program has an absolute minimum of harm associated with it. In reality, the point of minimal-sufficient-harm is a vague point and the main cause for IACUC discussions [22]. IACUCs strive to reach this minimal harm but there is always a zone left for discussions and changes. I argue that when adding HBA on top of the refinement principle, it is this gray zone that is affected and can change animal welfare, sometimes in significant ways. 

In an environment supporting HBA, the argument that a harmful procedure is justified because the research is translational and has the potential to save human lives is taken into account in the discussions, in addition to the argument that the procedure is the least harmful procedure necessary to achieve the goal (the 3Rs). The problem is that in some cases the two arguments conflict. In a way, built-in tension prevails between HBA and the 3Rs. For example, imagine a research study aimed at examining the therapeutic effects of a new drug that involves two options: one is to use a full-blown animal model of the disease allowing severe symptoms to develop; the other is to use a reduced animal model of the same disease that does not reach severe symptoms. According to the principle of Refinement, the latter model should be used because the refined model is adequate for teaching about the therapeutic potential of the drug. However, using the former model has the potential of increased benefit because it might demonstrate a significant improvement in severe symptoms. Thus, from the point of view of the benefit of the research, the former model is desirable and HBA, in this case, can lean the decision towards the more severe animal model. 

An additional likely outcome of HBA is an increased pressure on researchers to come up with research projects that can be easily presented as carrying important benefits. Such research projects, more often, require harmful experiments. Thus, HBA, indirectly and in unmeasurable ways, can lead to an increase of the overall severity of the research experiments for reasons that are not scientifically driven.

Again, these are not negligible theoretical concerns. In reality, IACUC members experience the pressure of accepting severe research procedures when the research is considered very important. I believe the HBA jargon commonly used in the context of animal experimentation can encourage the ease of demands in such research programs.

## 4. The Alternative Guideline to HBA 

Given the above caveats of HBA, I call policy-makers to reconsider the place of HBA in animal research. Instead, I support an alternative guideline that in my opinion addresses the concerns of animal welfare without the need to evaluate the benefits of the research.

The principle of HBA is that the benefits of the research must exceed the harm caused to the animals. The alternative is to replace this principle with two guiding principles:

1. Any research using an animal must carry a benefit for society.
2. Harm inflicted to the animals in the research must be minimal and scientifically justified.

The task of national and institutional committees then involves guaranteeing that these principles are not violated. Ensuring the first principle is mostly straightforward. We usually accept that research performed in an academic environment is conducted according to the rules of the scientific method, including a strong motivation to publish the research results (however, see a discussion of exceptional examples below). Research meeting these criteria carries the benefit of acquiring and distributing new knowledge. Thus, only researchers who are affiliated with accredited research institutes should be permitted to submit requests for carrying out animal experiments. On top of this, the feasibility and the quality of the research program (not the aims) can and should be evaluated by the committees as an additional way to strengthen the first guiding principle. 

Fulfilling principle 2 then becomes the major focus of IACUC operation by safeguarding proper anesthesia, proper analgesia, enrichment, adequate caging conditions, stress relief, proper diets, humane end-points and so forth. Ideally, ethical committees should concentrate efforts in a harm-analysis alone, striving to eliminate suffering *independent of the research question.*

But is relying on the scientific integrity of researchers affiliated with accredited institutes enough? Recall that the public concern is that scientists will waste animal lives for no good reason. If we do not evaluate the benefit, how can we guarantee that no scientist will present a research question that is unsound? I believe that this could be taken care of if we modify the common definition of the first R—Replacement:

“The term includes absolute replacements (i.e., replacing animals with inanimate systems such as computer programs) as well as relative replacements (i.e., replacing animals such as vertebrates with animals that are lower on the phylogenetic scale).”

NRC guide for the care and use of laboratory animals (8th edition)

The Replacement principle should be modified to include not only the replacement of the animal model to the lowest possible model but also the replacement of the scientific question to the most basic unstudied level. Science evolves in small steps, with each research study resting on results from previous more basic research studies. 

The diagram in Figure 1B illustrates the desired evaluation procedure. In this scheme, the scientific program is evaluated according to the three Rs. In parallel, the scientific question is also evaluated, however, not for its potential benefit (an impossible task) but for its theoretical background (a possible task). A scientific question is expected to rest on a solid theoretical background achieved in previous scientific studies. If not, according to the ethical principle of “Replacement,” it would not justify the use of animals to study it.

To understand how this works, consider the following example: a researcher requests permission to carry out an experiment to examine the effects of listening to classical music on the brain of mice and for this, he is requesting to expose mice to high-volume classical music 12 h a day and after three weeks to sacrifice the mice to examine their brains. The first impression is that this seems like an odd scientific question. But the scientist is entitled to academic freedom and we have no mandate to prevent him in pursuing his goals. The solution then comes from the ethical principle that one cannot jump directly to carry out invasive animal experiments. First, a theoretical foundation must be established for the question at hand. The scientist needs to present a research hypothesis that is rooted in previous scientific knowledge. Thus, the researcher needs to provide the committee with convincing evidence that classical music (and not other sounds) is expected to induce the specific changes in the brain. In other words, he must provide the theoretical background leading to predictions that would justify studying this scientific question. If such a background is not provided, the researcher must replace the research question to address a more basic level, for example: does environmental acoustic noise affect foraging behaviors in mice? The latter question is more likely to have a theoretical justification and answering it certainly requires less harmful procedures. 

## 5. Summary and Conclusions

In this short commentary I pointed to the inherited difficulties of the use of HBA in the evaluation and approval process of animal research. The difficulty arises from the nature of basic research where the benefits are manifested indirectly at a global level while the harms, if exist, are inflicted directly at the individual research level. The utilitarian principle asserts that the global benefits of biomedical research should overcome the total harms. I believe and argue above, that the best way to achieve this goal is to separate the harm analysis from the benefit analysis. Moreover, I demonstrate how rigorous harm analysis alone may rule out research programs that are not scientifically justified. 

Over the years, numerous and lengthy discussions of HBA in animal research have been conducted in an attempt to provide clear and general rules for HBA [2,3,12,13,14]. Maybe, instead of continuing to struggle with the difficulties, it is time to reconsider the use of HBA at the individual research program and to explore alternative guidelines.
